# The Small World of Psychopathology

**DOI:** 10.1371/journal.pone.0027407

**Published:** 2011-11-17

**Authors:** Denny Borsboom, Angélique O. J. Cramer, Verena D. Schmittmann, Sacha Epskamp, Lourens J. Waldorp

**Affiliations:** Department of Psychology, University of Amsterdam, Amsterdam, The Netherlands; Georgetown University Medical Center, United States of America

## Abstract

**Background:**

Mental disorders are highly comorbid: people having one disorder are likely to have another as well. We explain empirical comorbidity patterns based on a network model of psychiatric symptoms, derived from an analysis of symptom overlap in the Diagnostic and Statistical Manual of Mental Disorders-IV (DSM-IV).

**Principal Findings:**

We show that a) half of the symptoms in the DSM-IV network are connected, b) the architecture of these connections conforms to a small world structure, featuring a high degree of clustering but a short average path length, and c) distances between disorders in this structure predict empirical comorbidity rates. Network simulations of Major Depressive Episode and Generalized Anxiety Disorder show that the model faithfully reproduces empirical population statistics for these disorders.

**Conclusions:**

In the network model, mental disorders are inherently complex. This explains the limited successes of genetic, neuroscientific, and etiological approaches to unravel their causes. We outline a psychosystems approach to investigate the structure and dynamics of mental disorders.

## Introduction

One of the major discoveries in the past century of psychopathology research is that *comorbidity* (the joint occurrence of two or more mental disorders) is the rule rather than the exception [1]. Since comorbidity has consistently been associated with a poorer prognosis and greater demands for professional help, unraveling the causes of comorbidity ranks among the top priorities in clinical psychology and psychiatry [2], [3].

In practice, comorbidity is often investigated by analyzing the association between composite measures defined on two sets of items (i.e., a correlation between total scores on checklists) or between two diagnoses. This methodology has yielded important insights into which disorders co-occur more frequently than chance [1], [4], [5] and into risk factors that predispose towards comorbidity [6]–[8].

However, a limitation of this methodology is that symptoms are viewed as passive indicators of “latent” conditions (disorders) that do the actual causal work [9], [10]. Comorbidity is then suggested to arise from a root cause that is shared by two or more latent disorders. For instance, shared genes or a general predisposition towards negative affect have been put forward as common causes of comorbidity between Major Depressive Episode (MDE) and Generalized Anxiety Disorder (GAD; [11], [12]).

In clinical psychology and psychiatry, however, symptoms are unlikely to be merely passive psychometric indicators of latent conditions; rather, they indicate properties with autonomous causal relevance. That is, when symptoms arise, they can cause other symptoms on their own. For instance, among the symptoms of MDE we find *sleep deprivation* and *concentration problems*, while GAD comprises *irritability* and *fatigue* [13]. It is feasible that comorbidity between MDE and GAD arises from causal chains of directly related symptoms; e.g., *sleep deprivation* (MDE)→*fatigue* (MDE)→*concentration problems* (GAD)→*irritability* (GAD). For other disorders, such chains appear plausible as well: e.g., *having suffered from a panic attack* (panic disorder)→*being worried about having another attack* (panic disorder)→*avoiding public places* (agoraphobia). In accordance with this idea, Kim and Ahn [14] showed that clinical psychologists typically interpret symptom patterns in terms of causal networks.

**Figure 1:**
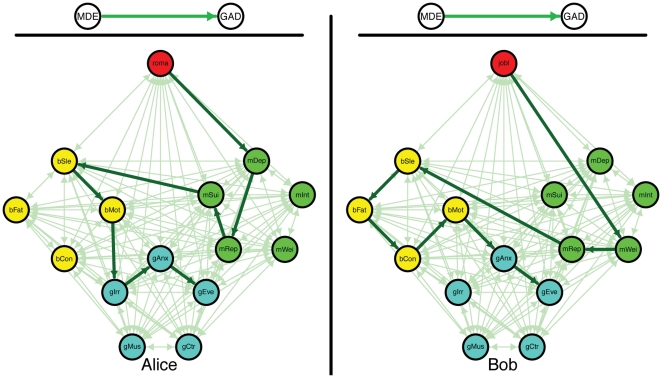
The difference between the existing view on comorbidity (*top*) versus the network approach (*bottom*) for two fictitious persons, Alice (*left*) and Bob (*right*). In both figures, the *red node* represents an external life event; *green nodes* core MDE symptoms; *turquoise nodes* core GAD symptoms; *purple nodes* bridge symptoms (i.e., symptoms that are part of both MDE and GAD). Edges between nodes represent pathways between symptoms. The light green edges represent possible pathways; the thicker and dark green edges the pathways taken by Alice and Bob respectively. *roma* = break-up of romantic relationship; *jobl* = job loss; *mWei* = weight problems; *mInt* = loss of interest; *mRep* = self-reproach; *mDep* = depressed mood; *mSui* = (thoughts of) suicide; *bSle* = sleep problems; *bFat* = fatigue; *bCon* = concentration problems; *bMot* = psychomotor problems; *gAnx* = chronic anxiety; *gEve* = anxiety about more than one event; *gCtr* = no control over anxiety; *gMus* = muscle tension; *gIrr* = irritable.

Thus, it is likely that direct relations between symptoms exist. As a result, there may be individual differences in how comorbid disorders develop. There may not just be *one* source of comorbidity, for which it does not matter which symptoms are in effect for a given person; there may instead be *many* roads to comorbidity, and which one is taken depends on the person and his or her specific situation. Figure 1 shows an illustration of this idea, by highlighting possible roads to comorbidity between MDE and GAD for two fictitious persons, Alice and Bob. In both, MDE leads to GAD, but in different ways and for different reasons. For instance, Alice may develop depressed feelings due to the break-up of a romantic relationship, while Bob may become overweight after losing his job (see Figure 1). Such differences are plausible, because the onset of symptoms of mental disorders like MDE is differentially related to distinct life events [15]. In addition, the events that set the domino effect in motion may affect the subsequent road that is taken to comorbidity (Figure 1: Alice and Bob travel different roads but both end up with a MDE and GAD diagnosis).

Reasoning along these lines, our research team has developed a *network* approach to mental disorders and comorbidity [16]. In this approach, symptoms are not viewed as indicators of latent conditions, but as components in a network; comorbidity is hypothesized to result from direct relations between symptoms of multiple disorders. Here, we use this approach to analyze the entire symptom space described in the Diagnostic and Statistical Manual of Mental Disorders-IV (DSM-IV; [13]). We construct a network of this space and show that (a) half of the symptoms in the DSM-IV are directly or indirectly connected in a so-called *giant component*, and (b) this component of connected symptoms has the characteristics of a *small world* [17]. This means that a significant part of DSM-IV comorbidity may indeed arise through the effects of symptoms shared by multiple disorders (i.e., *bridge symptoms*; [16]). In addition, we show through simulations that the network model can account for population statistics on prevalence and comorbidity.

## Results

### Study 1: Construction and analysis of the DSM-IV-network

A first step towards providing an account of comorbidity from a network perspective is to examine the most often used taxonomy of mental disorders, as represented in the DSM-IV. More specifically, to get a first approximation of the symptom space, we aim to construct a network that represents all individual DSM-IV symptoms and connections between them. Subsequently, we analyze its global structure by the application of network analysis techniques [18].

#### Graph construction

The DSM-IV lists 201 distinct mental disorders, which are diagnosed through 522 criteria. Diagnostic criteria do not map onto symptoms uniquely. Sometimes the same symptoms function as criteria for distinct disorders (e.g., *fatigue* is a symptom of MDE and of GAD), sometimes one symptom is a special case of another symptom (e.g., *insomnia* is a special case of *sleep disturbance*), and sometimes two symptoms only differ with respect to their antecedent causes (e.g., *insomnia* versus *insomnia due to alcohol withdrawal*). We dealt with this overlap by a) equating symptoms that are literally the same or that are so similar that they are unlikely to be behaviorally distinguishable (e.g., *restlessness* in GAD and *psychomotor agitation* in MDE), b) discounting symptoms that refer to external influences (e.g., when *insomnia* is already present, *insomnia due to alcohol withdrawal* is not represented as a distinct symptom), and c) separating disjunctive symptoms into their constituent parts (e.g., *insomnia or hypersomnia*, a criterion for MDE, is decomposed into *insomnia* and *hypersomnia*). The resulting 439 symptoms were used for further analysis.

As a result of this methodology, disorders that feature exactly the same symptoms (e.g., *Bipolar I Disorder, Most Recent Episode Hypomanic* versus *Bipolar I Disorder, Most Recent Episode Manic*) are no longer represented as distinct entities. The same holds for disorders that only differ by reference to external precipitating factors (*Delirium Due to a General Medical Condition* versus *Substance Withdrawal Delirium*). As a result, the number of explicitly represented disorders in the final analysis (148) is smaller than the number in the DSM-IV (201). The symptoms and their allocation to disorders are available from http://www.psychosystems.org/materials.html.

The network was constructed as follows. First, we represent each of the DSM-IV-symptoms as a *node*. Second, we draw a connection (i.e., an *edge*) between any two symptoms if they function as criteria for the same disorder. These two steps define a *network structure* among the symptoms. Technically this may be viewed as a projection of a bipartite network, in which symptoms are connected via common disorders, onto the symptom space. In the resulting network structure, symptoms of the same disorder become directly connected, while indirect connections arise because of the presence of shared symptoms (i.e., bridge symptoms). For instance, *insomnia* and *lack of interest* are directly connected, because both are symptoms of MDE. *Lack of interest* and *anxiety*, in contrast, are not directly connected (because there are no DSM-IV disorders that feature both of these symptoms). However, these symptoms are connected indirectly via *insomnia*, because *insomnia* is a shared symptom of MDE (which also has *lack of interest* among its symptoms) and GAD (which includes *anxiety* among its symptoms). Finally, two symptoms are unconnected if there is no way of getting from one to the other via bridge symptoms. To visualize this network structure, we employed a node positioning algorithm with the R-package igraph [19] which leads more strongly connected sets of nodes to cluster closer together [20].

#### Network analysis

**Figure 2:**
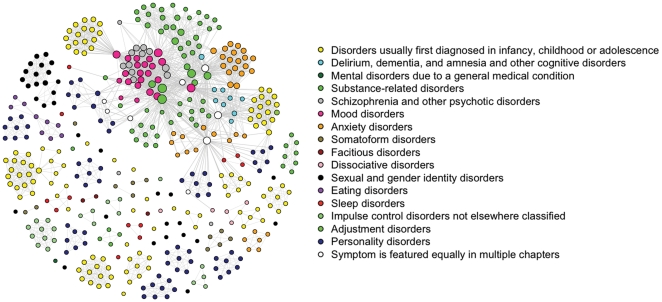
The DSM-IV symptom space. Symptoms are represented as nodes and connected by an edge whenever they figure in the same disorder. Color of nodes represents the DSM-IV chapter in which they occur most often.

Figure 2 shows the resulting DSM-IV symptom graph (to identify individual symptoms as well as the symptom-disorder correspondence, consult http://www.psychosystems.org/Data/Pics/Graphs/DSMgraph.svg with a browser that can show .svg files, such as Firefox). The graph has two properties that are interesting in view of the generally high comorbidity between DSM-IV diagnoses. First, it features a *giant component* spanning 208 (47.4%) of the symptoms: roughly half of the symptoms are connected. This means that one can reach any of these symptoms from any other, by traveling along a path consisting of one or more edges between them. Second, the structure of the giant component conforms to a *small world* [17]. This means that, compared to a graph in which the same number of nodes (208) is connected by same number of edges (1949) in a completely random fashion (a *random graph*) it has a similar short path length between symptoms, but a much higher probability of an edge between two symptoms that are direct neighbors of a third symptom, i.e., a higher level of clustering. Table 1 gives an overview of the characteristics of the giant component in the DSM-IV network.

**Table 1:** Properties of the DSM-IV giant component.

Global properties	
Number of symptoms	208
Number of explicitly represented disorders	69
Number of edges	1949
Average shortest path length	2.60
Average number of shortest paths between two symptoms	3.01
Small-worldness index (SWI), based on transitivity	6.20
Clustering coefficient, based on transitivity	0.68
Average degree	18.74

Symptoms with highest degrees	
Symptom name	Degree	% Connected symptoms
1. Insomnia	71	34.1%
2. Psychomotor agitation	68	32.7%
3. Psychomotor retardation	61	29.3%
4. Depressed	60	28.8%

Symptoms with highest random walk betweenness	
Symptom name	Betweenness	% Connected symptoms
1. Irritable	0.24	23.6%
2. Distracted	0.17	24.0%
3. Anxious	0.16	23.1%
4. Depressed	0.16	28.8%

**Figure 3:**
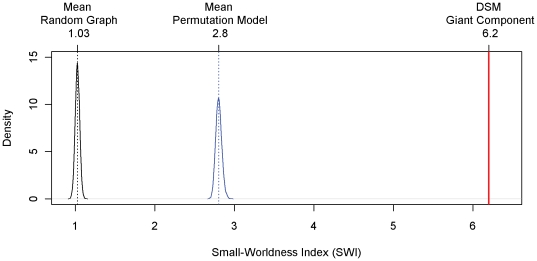
Small-world-ness indices (SWI). Density distributions of the SWI's of 10000 random networks (in black), and of 10000 permutation model networks (in blue). The vertical red line marks the observed SWI of the giant component. Dotted vertical lines indicate the respective mean SWI.

To establish the small world property, we examined the clustering coefficient and average shortest path length of the DSM-IV graph. The clustering coefficient, based on transitivity, is defined as 3 times the number of triangles divided by the number of paths of length 2 [21]. The shortest path length ℓ(i,j) between two nodes i and j equals the minimum number of edges that must be passed over to get from i to j. The average shortest path length L is the average over the shortest path lengths ℓ(i,j) of all node pairs [22]. With a clustering coefficient of C_DSM_ = 0.68 and an average shortest path length of L_DSM_ = 2.6 (as opposed to the expected values of C_RND_ = 0.09 and L_RND_ = 2.12 in a random graph; [22]), the giant component of the DSM-IV network has a small-world-ness index (SWI = (C_DSM_/C_RND_)/(L_DSM_/L_RND_); [21]) of 6.2, which exceeds the conservative small-world-ness criterion threshold of 3 [21]. Figure 3 shows the density of the SWIs of 10000 random networks (in black), along with the observed SWI of the giant component (red line). The assortation of symptoms and disorders that underlies the giant component differs from a random assortation schedule with respect to small-world-ness in the resulting symptom networks. To test this, we determined the SWIs of 10000 networks, in which 208 symptoms were assorted randomly with the 69 disorders in the giant component, while each symptom and each disorder had exactly the same number of connections as in the giant component (henceforth: permutation model).

Figure 3 shows the density of the SWIs of the simulated permutation model networks (in blue), which is located in between, and is well separated from, the density of the random network SWI's and the SWI of the DSM giant component. In addition, the probability p of a connection between any two given symptoms is larger in these networks (0.15<p<0.17) than in the DSM giant component (p = 0.09). This shows that in the DSM giant component, symptom groups figure together in multiple disorders more often than expected under a random assortation schedule. This is exactly what we would expect if symptom groups are causally related. In sum, the existent giant component, and its small world characteristic, reflect that half of the symptoms are indeed connected, and that the paths to comorbidity arising from these connections are typically short, with symptom groups figuring together in multiple disorders.

**Figure 4:**
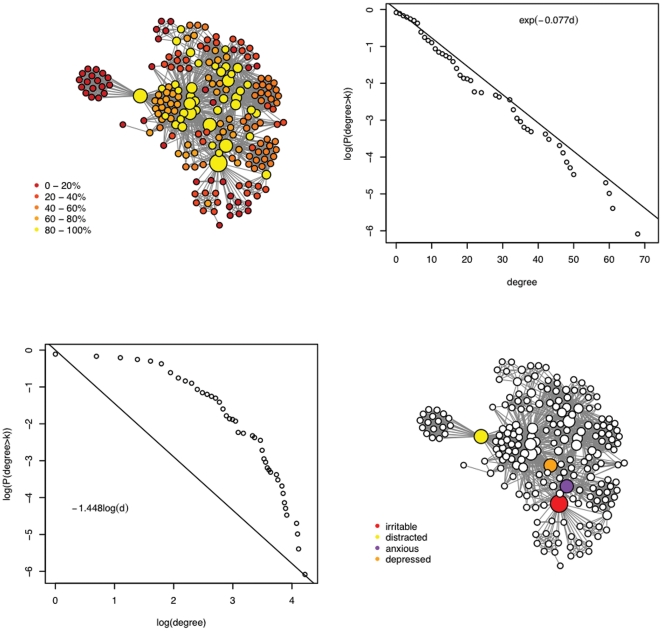
The giant component and its degree distribution. In the top left and bottom right parts of the figure, node size is proportional to the centrality of the node: the more central, the larger the node. The represented centrality measure is based on *random walk betweenness* [36]. That is, we averaged the number of times that a node was part of a path between two other nodes chosen during consecutive random walks. The top left part represents the giant component while the bottom right highlights the four most central symptoms. The top right and bottom left parts of the figure show the fit of two functions on the degree distribution: logistic (to assess power law property; left bottom) and exponential (to assess exponential decay; top right). The x-as represents the (log) degree while the y-axis represents the probability that a node chosen uniformly at random has a degree larger than *k*.

An important property of nodes in any network is their centrality [18]. Two of the standard ways of measuring this property are by determining the *degree* and *betweenness* of nodes. The *degree* of a node equals the number of connections a node has with other nodes. Nodes with a higher degree thus are more central in the sense of having more direct connections. The *degree distribution* (i.e., the probability that a node selected at random has a certain degree) of a network gives important information about network structure and is often used in network analysis to classify networks [23]. The degree distribution of the DSM-network follows an exponential decay function (top right Figure 4) rather than a power law (bottom left Figure 4), which classifies it as a *single-scale network* [23]. This means that the DSM-IV network is of the same type as, for instance, an air-traffic network with airports (nodes) that are connected to small to large numbers of other nodes, yet without nodes that are connected to extremely large numbers of other nodes [23]. Similarly, the four most highly connected symptoms of the DSM-IV network are linked to 60 to 71 other symptoms (i.e., 14–16% of all symptoms; 29–35% of symptoms in the giant component). The symptom with the highest degree is *insomnia* (71 connections), followed by *psychomotor agitation* (68), *psychomotor retardation* (61) and *depressed mood* (60). Another important property of nodes that is relevant in thinking about comorbidity is their betweenness. This measures the probability that a node lies on a path between two other nodes. In the DSM-IV graph, the four symptoms with the highest (random walk) betweenness are *irritable*, *distracted*, *anxious* and *depressed* (bottom right Figure 4).

Symptoms with a high centrality may transmit the effects from many symptoms to many other symptoms. The most central symptoms in the DSM-IV network are rather ordinary, in the sense that they are commonly experienced in response to a variety of external events, such as a quarrel with a loved one or failure to pass an exam. However, they are also likely to arise from conditions within the DSM-network. Such diverse conditions as alcohol addiction, lacking close friends, or amnesia, may trigger any of these central nodes; in turn, when activated, their effects may fan out across the network. Thus, the DSM-IV network suggests that there are many short roads to comorbidity, and more often than not, these run through what we may consider “mundane” symptoms. Interestingly, the most central symptoms in the DSM-IV network all function as criteria for two of the mental disorders with the highest prevalence in the US, namely mood and anxiety disorders [24]. This is consistent with the idea that symptom activation indeed spreads though the network structure along the paths represented in Figure 2.

### Study 2: Connecting network structure to empirical data

To the extent that the network as depicted in Figure 2 approximates the causal structure of DSM-IV symptoms adequately, the small world property implies that symptom activation will spread rapidly through the network, analogous to the way an epidemic spreads through a population [25]. Thus, the network model potentially explains a significant part of the substantial comorbidity among DSM-IV categories. Further, if we make some basic assumptions about this process, we can derive empirical predictions from the network hypothesis at the level of the global comorbidity structure of the DSM-IV. For instance, we may plausibly assume that the majority of symptoms in the DSM-IV network are positively connected; that is, having any DSM-IV symptom results in a *higher* probability of developing another one. As such, we assume that symptoms do not function as protective factors in the development of other symptoms (i.e., resulting in a *lower* probability). If this assumption is correct, we should expect only positive empirical correlations between mental disorders (a *positive manifold*). Second, we should expect an association between distances between disorders in the network and empirical comorbidity rates: the farther apart two disorders are, the lower the probability of comorbidity between them.

#### Average shortest path lengths and comorbidity

To examine these network predictions, we first determined the distance between disorders in the graph. For this purpose, we used the shortest path lengths^2^ between symptoms. The distance between two disorders was then operationalized as the average shortest path length between their respective symptoms. The average shortest path length between two disorders A and B is thus equal to the expected number of edges one has to travel to reach a randomly chosen symptom from disorder A from a randomly chosen symptom from disorder B. The higher this number is, the farther apart the disorders in question are situated in the network.

To relate these statistics to empirical comorbidity rates, we used existing reports on the comorbidity structure in the National Comorbidity Survey Replication (NCS-R; [1], [4]), in which a sample of over 9,000 individuals completed a structured DSM interview. In particular, we used the tetrachoric correlations between common mental disorders as reported in Table 1 of Krueger's [26] analysis as our operationalization of comorbidity. Subsequently, we related the average shortest path lengths between disorders to the empirical comorbidity rates. Two of the disorders discussed in [26], *Substance Dependence* and *Substance Abuse*, are not part of the giant component; hence, we could not include them in this analysis. However, note that although there are no bridge symptoms that connect these disorders with, e.g., major depression, it is likely that causal relations between these disorders do exist.

**Figure 5:**
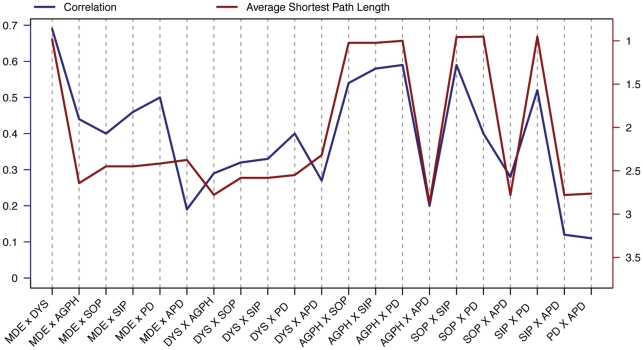
Average shortest path length and comorbidity. Left y-axis represents comorbidity; right y-axis average shortest path length. Abbreviations: *MDE* = Major Depressive Episode; *DYS* = Dysthymia; *AGPH* = Agoraphobia; *SOP* = Social Phobia; *SIP* = Simple Phobia; *GAD* = Generalized Anxiety Disorder; *APD* = Antisocial Personality Disorder.

Figure 5 shows the empirical correlations between 28 pairs of common DSM-IV disorders (the blue line; [26]) and the corresponding average shortest path lengths between the symptoms that make up these disorders in our graph (the red line). As is evident from the figure, the correlation between empirical comorbidity rates and average shortest path lengths is substantially negative (*r* = −0.72 over all pairs of disorders; *r* = −0.66 for pairs of disorders that have no common symptoms). In addition, correlations between disorders are never negative (i.e., they form a positive manifold), consistent with the network model.

Naturally, this does not constitute proof of the model. First, the data are consistent with our hypothesis that the causal connections between symptoms produce the correlations in question, but we cannot rule out the possibility that other sources of comorbidity led to this pattern by happenstance. This is relevant because the DSM-IV itself was constructed partly on the basis of empirical correlations (i.e., it is likely that the scholars constructing the various editions of the DSM used the correlations between symptoms to construct syndromes). In addition, the disorders themselves partly result from arbitrary or purely pragmatic decisions. Hence the structure of the graph is unlikely to be definitive. Finally, although common mental disorders cover a large share of psychopathology in terms of prevalence, the number of disorders that we could include in the analysis is limited. Keeping in mind these caveats, however, the results could easily have panned out differently, as there is no statistical law that prescribes that disorders constructed in the DSM-IV fashion should correlate positively, or that the relation between average shortest path lengths and empirical correlations should be negative. Thus, in our view, the results have some evidential relevance, if only because they show that a network explanation of the global DSM-IV comorbidity structure is feasible.

### Study 3: Simulating symptom dynamics of MDE and GAD

The previous analysis shows that path lengths and empirical comorbidity rates are correlated as would be expected if symptom activation propagates via the network structure. However, disorders are usually temporally dynamic, in the sense that they are defined with respect to certain time frames (e.g., two weeks for MDE and six months for GAD). Thus, one would ideally demonstrate that the dynamic properties of the network can account accurately for empirical comorbidity rates as they would arise in time.

**Figure 6:**
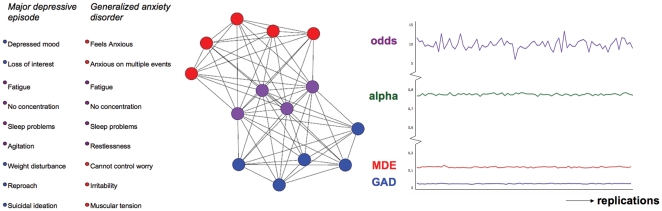
The simulation of MDE and GAD and its results. The left part of the figure shows core MDE (*blue nodes*), core GAD (*red nodes*) and bridge symptoms (*purple nodes*). The middle part of the figure represents the implied structure of the simulated network: comorbidity arises through connections via bridge symptoms. There are no direct connections between core MDE and core GAD symptoms. The right part of the figure displays the results of the simulations. The x-axis represents the number of replications of the simulation. The y-axis represents 1) *odds*: odds ratio of diagnoses as measure of comorbidity, 2) *alpha*: Cronbach's α, 3) *MDE*: prevalence of MDE and 4) *GAD*: prevalence of GAD.

The network model is potentially capable of this; when coupled with a temporal regime that specifies the causal influence of nodes on each other, the model becomes a dynamic and intra-individual model that describes how mental disorders and comorbidity between them develop over time in individual persons (like the networks for Alice and Bob in Figure 1). As such, the edges between symptoms represent mediational processes (e.g., homeostatic and cognitive processes) that, triggered by having symptom A at time point 1, could result in symptom B at time point 2. For instance, anxiety (symptom A) is likely to trigger ruminative processes that result in depressed feelings (symptom B). Now, one way to demonstrate the potential of the network approach to predict empirical comorbidity rates is to simulate dynamics implied by a network for two disorders and to show that the resulting prevalence and comorbidity rates are consistent with their empirical counterparts. This is what we did for two disorders with a high comorbidity rate: MDE and GAD. Figure 6 shows that we simulated data on 14 symptoms for which comorbidity arises only through pathways that include bridge symptoms (i.e., no core MDE symptoms are directly connected with core GAD symptoms and vice versa).

#### Simulation setup

We specified the symptom dynamics as follows: for a given symptom in the network, the probability of occurrence increases monotonically with the number of neighboring symptoms that are activated. For instance, if someone suffers from depressed mood and loss of interest, the probability of developing thoughts of suicide is higher than for someone who suffers from loss of interest but does not suffer from depressed mood. The parameters of the network symptoms were derived from an analysis of the NCS-R data. We then simulated the development of MDE and GAD networks for 9282 fictitious persons over 365 time points (“days”), allowing for the reconstruction of yearly prevalence and comorbidity rates for comparison against benchmark NCS-R data (which have the same number of persons).

The simulation details are as follows. At time *t*, a node *i* has a probability *p_it_* of turning ‘on’ (taking value 1) and of 1-*p_it_* of turning ‘off’ (taking value 0). The probability *p_it_* is determined by the logistic function *p_it_ = a*+(1−*a*)[exp{*b_i_A_i_*_(*t*-1)_−*c_i_*}/(1+exp{*b_i_A_i_*_(*t*-1)_−*c_i_*})−*a*], where *A_i_*_(*t*-1)_ is the number of neighbors of *i* that are activated at *t*-1, *b_i_* is the sensitivity of symptom *i* to that activation sum, and *c_i_* is a symptom specific threshold. We chose this probability function because it has the plausible property of monotonicity and because it is similar to the response function of common Item Response Theory (IRT) models that are often used in clinical psychology [10]; hence we anticipated that this function would produce data that has psychometrically realistic properties. In addition, the parameters of the logistic function can be based on the analysis of available data, which allows us to use empirical values for the network parameters. In particular, we derived the *b_i_* and *c_i_* parameters from logistic regression analyses executed on the NCS-R data, where (dichotomous) symptom endorsement was regressed on the total number of symptoms endorsed for the disorder in question; *b_i_* and *c_i_* are respectively the slope and location parameters of that regression. Finally, the parameter *a* controls the base rate probability that a symptom will activate even if no neighbours are on, and the recovery rate probability that a symptom will deactivate even if all of its neighbors are on. As the network architecture is derived from the DSM-IV, and the *b_i_* and *c_i_* parameters from the NCS-R data, *a* is the only free parameter in the simulation. Setting *a* to a value of .22 produced reasonable results (this effectively corresponds to a base rate of .05 and a recovery rate of .18) and was used in the simulations reported. Simulation code is available from http://www.psychosystems.org/materials.html.

After a simulated “year”, we “diagnose” each network for presence of MDE and/or GAD according to DSM-IV criteria. We do this by examining which symptoms were present at which time points; e.g., when a network featured activation of five out of nine MDE symptoms for a period of at least fourteen days, and these included at least one of the core symptoms *depressed mood* and *loss of interest*, the network was diagnosed with MDE. For GAD, most of the symptoms had to be present for most of the days in at least six months (182 time points), including the core symptoms *anxious*, *worried about multiple events*, and *failure to control the worries*. Finally, we compare population statistics to those known from empirical research. As a conservative check for specificity of the results, we also obtained results from a random model that had the same network architecture as the original model, but in which the pairs of parameter values (*b_i_, c_i_*) from the NCS-R data were assigned randomly to the symptoms. To check for stability of the results, we replicated the original model and the random model simulation 1000 times each.

#### Simulation results

Figure 6 shows that the simulated population of networks yielded stable and empirically plausible results. The means for disorder prevalence over simulations are .12 for MDE and .02 for GAD, which corresponds reasonably with empirically established prevalence rates (around .1 for MDE and .03 for GAD; [1], [4]). Comorbidity between MDE and GAD as assessed by the odds ratio of diagnoses is 10.08, which is somewhat too high, but in our view still lies within the bounds of empirical plausibility (e.g., in the NCS-R data the odds ratio lies around 7; [27]). In a traditional psychometric analysis, the symptoms would yield a mean(SD) Cronbach's alpha of .78(.005) over all symptoms, as measured at the last time point of each simulation. We also fitted a two parameter logistic model [28], which is known to fit the MDE symptoms [29], to 100 slices of data, each of which represents a single timepoint in a simulation. Although the parameter estimates for the simulations are not directly comparable to those of the NCS-R data due to the complicated structure of the NCS-R interview, the correspondence is still reasonable; the mean(SD) correlation between the estimated difficulty parameters in the simulation and in the NCS data equals .70(0.01); this value is .61(.03) for the discriminations. In all, the patterns in the simulated data do not appear to strongly conflict with the empirical patterns we see in the NCS data.

A noteworthy result is that GAD symptoms were, on average, more prevalent than MDE symptoms: the lower prevalence of GAD was solely produced by the stringent duration criterion - i.e., in GAD, symptoms need to be present for most days in the last six months, whereas MDE only requires symptoms to be present for the last two weeks. The same is true in the NCS-R data.

**Figure 7:**
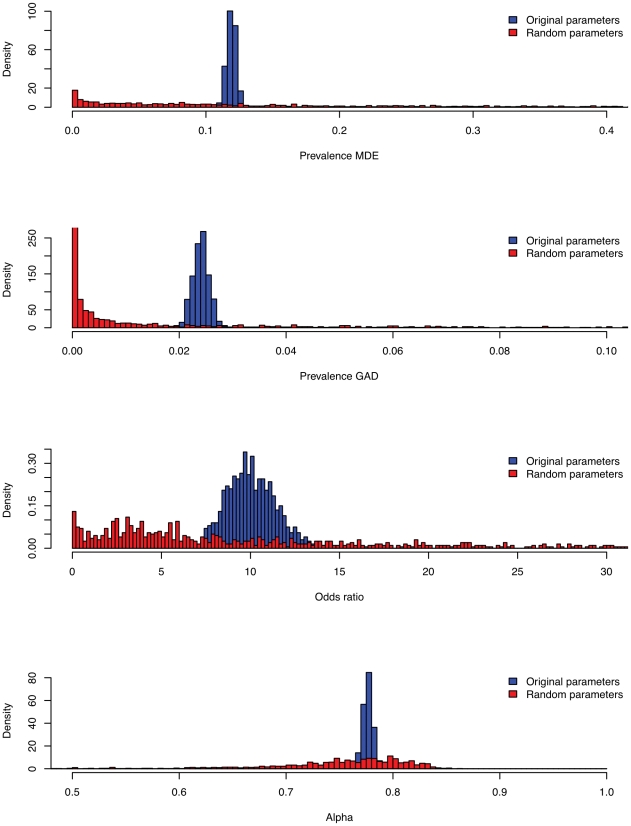
Densities of simulation results of original vs random parameter values. Top to bottom: Prevalence of MDE, Prevalence of GAD, Odds ratio, Cronbach' alpha. Densities of networks resulting from original (random) parameter values are shown in blue (red).

The question arises whether the empirical plausibility of the results depends on the use of appropriate empirically derived parameter values, or can be ascribed merely to the structure of the network model (independent of its parameterization). To investigate this, we compared our results to the random model replications. To facilitate this comparison, we heuristically defined empirically plausible ranges (see Table 2) to be in broad agreement with the empirical literature. Table 2 shows the percentages of original and random model replications that gave plausible results for MDE and GAD prevalences, odds ratio, and Cronbachs alpha. A total of 99.9% of the original model replications resulted in overall plausible results (i.e., models which jointly satified the empirically plausible ranges for all variables), while this was the case for 3.2% of the random model replications. This difference does not depend substantially on our choice of empirically plausible ranges; this can also be seen from Figure 7, which shows the distribution of original and random model results over the outcome variables.

**Table 2:** Results of simulations.

	Plausible range	Mean (Sd.)		% replications in plausible range	
	Empirical	Random	Original	Random	Original
Prevalence MDE	0.05–0.15	0.199 (0.2193)	0.119 (0.004)	27.9	100
Prevalence GAD	0.01–0.05	0.059 (0.1465)	0.024 (0.005)	19.7	100
Odds ratio	5–15	40.390 (86.933)	10.081 (1.411)	28.2	99.9
Cronbach's Alpha	0.6–1	0.749 (0.068)	0.777 (0.005)	96	100
Overall				3.2	99.9

It should be noted that the results are derived under an extremely simple dynamical scheme. Because of this simplicity, several properties of the simulated networks are empirically implausible. For instance, there are no individual differences in network parameters, while it is likely that such differences do exist in reality. However, the points at which the model is implausible concern overly restrictive rather than lenient aspects of the model, and therefore work against rather than for the fit to empirical data. For instance, if individual differences were included in the simulations, e.g., by making network parameters random, the model would become more flexible, making it easier to match empirical data even better than the current model does. In fact, that such an austere setup of the network model can come so close to the actual data is perhaps the most unexpected and surprising feature of this work.

Thus, although the simulated networks are incomplete and idealized reflections of reality, the results do establish a proof of possibility by showing that the network model can produce empirically realistic results. Furthermore, these empirically realistic results emerged consistently if and only if appropriate empirically derived parameter values were used, that is, the results were specific to the parameterized network model rather than to network architecture alone. To the best of our knowledge, this network model is the first dynamical model of psychopathology that simultaneously explains population statistics regarding prevalence, comorbidity, and internal consistency, as found in studies like the NCS-R.

## Discussion

The cause of comorbidity is a puzzle, which may have its roots in the very conceptualization of what a mental disorder is [9], [16]. Although much has been learned about the genetics, neuroscience, and etiology of mental disorders, the past century of research also shows that they cannot be identified with a simple set of genetic antecedents, neural correlates, or developmental trajectories. The network hypothesis may be considered to provide an explanation for this situation.

First, consider genetics. Behavior genetic research has shown that individual differences in the liability to develop disorders are, for a large part, genetically determined (e.g., genes are estimated to be responsible for around 50 percent of the variance in the psychological traits considered in [30]). However, only a minor part of the genetic variance can typically be traced to identified polymorphisms (often less than 2 percent for psychological traits; [31], [32]). This puzzle is known as the problem of *missing heritability* [33]. In the present view, it is plausible that the strength of symptom connections in a network partly stands under genetic control, but it is unlikely that all connections in a disorder are affected by the same genes in all people. Instead, we may consider the heritability of mental disorders to arise from, e.g., the genetic transmission of intersymptom connection strength, which in turn determines global parameters of person specific networks that correspond to the vulnerability of the system with respect to external and internal shocks. If this is correct, the network model may provide an alternative explanation to missing heritability, as compared to the currently proposed hypothesis that the heritability of mental disorders result from very large numbers of polygenes with small additive effects [33]. Further research should evaluate which of these hypotheses is more plausible for which disorders.

Second, consider neuroscientific research strategies into mental disorders. In the public eye, such studies have appeared to reveal what, say, depression “really is” by linking such a disorder to, for instance, a neurotransmitter imbalance. However, even though neuroscientific research has provided a wealth of empirical information about the correlates of mental disorders, no simple identifications of disorders with neural dysfunctions have been forthcoming. The network hypothesis suggests that this will remain the case, because neural properties are most likely to enter the model as mechanistic realizations of nodes and edges already in the network, or as additional nodes and edges that extend it. Such properties may contribute to the network structure importantly: for instance, many of the symptoms in the DSM-IV relate to basic homeostatic brain functions (eating, sleeping, sex, mood regulation), and it is essential to investigate their precise role in sustaining the network structure. However, just like the small world properties of the World Wide Web do not reduce to physical properties of individual webservers, mental disorders are unlikely to correspond to a single, homogeneous neural substrate.

Third, consider etiology. Some researchers have proposed that a focus on etiology may lead to a homogeneous grouping of mental disorders. In our view, this is unlikely. The giant component in the DSM-IV topology features 208*(208-1)/2 = 21,528 pairs of symptoms. Even if we just count the number of distinct shortest paths between any two symptoms in this topology, we obtain 129,643 distinct pathways. Although many pathways in the DSM-IV symptom space are unlikely to be active in transmitting effects, and it is probable that some pathways are more prevalent than others, it would seem unlikely that a focus on etiology could bring this number down to manageable size. Instead, the network approach predicts that individual cases of mental disorders will be highly idiosyncratic, both in the genetic and environmental determinants of the disorder, as well as in the etiological pathway by which it developed. The interesting fact is that, under the network approach, this may be the case even though population statistics relating to mental disorders are empirically stable. In this sense, the network hypothesis simultaneously accommodates the stability of population statistics in this area of research, and the idiosyncratic unpredictability of the individual person.

Thus, the network model not only yields plausible explanations for characteristic patterns in empirical psychopathology data, as shown in the research reported here; it may also illuminate the limited successes of research paths that have so far been taken. In particular, if mental disorders correspond to networks of causally coupled variables, we should not expect them to conform to a homogeneous biological, genetic, or etiological analysis. Instead, similar to current systems approaches in biology [34], research into psychopathology may profitably adopt a *psychosystems approach* by investigating the inherent complexity of mental disorders [35] through explicit models of the interplay between their psychological, biological, and social features that play a role in the development of psychiatric conditions, understood as clusters of causally linked properties [37], [38].

Further research in this direction may be pursued along the following lines. First, it is important to augment the network structure with “positive” nodes, that is, with nodes that are known to act as protective factors against developing pathologies (e.g., coping mechanisms used to “ward off” symptoms, or situational factors that may protect certain symptoms from rising to the level of pathology). This requires the construction of an “inverse” DSM, i.e., a categorized list of anti-symptoms that protect against disorders by blocking the spreading of problems through the network. Second, research into the direction and nature of the causal effects between network nodes (i.e., symptoms and anti-symptoms) should lead to a more refined representation of the network structure. Third, it is of interest to investigate how traditional measurement models, which for instance feature higher-order factors like internalizing and externalizing [26], relate to network structures. In traditional models, these factors are most readily interpreted as latent common causes of the item responses [10], [39], [40], but in the network models considered here, such common causes are lacking. Our expectation is that the relevant factors can be viewed as approximately isomorphic to regions of strongly connected symptoms, but whether this is conceptually and mathematically tenable is a question for further research. Fourth, it is important to test the dynamics of the network model against real data. Experience sampling studies, or other ways to track symptom dynamics, would appear to be especially suited for this purpose. Such research could also begin to unravel intra- and interindividual differences in network structure, which would open the possibility to examine salient network characteristics of individuals known to be at risk for developing mental disorders. If such characteristics could be charted, specific targeting of the most important network components (either with medication or with psychotherapy) might offer a novel way to develop therapeutic interventions and monitor their effects.
